# Effects of Thermal Pretreatment and Spontaneous Fermentation on the Quality Characteristics of Okara Flour

**DOI:** 10.1002/fsn3.72453

**Published:** 2026-10-04

**Authors:** Abdul‐Aziz Bawa, Fortune Akabanda, Newlove Akowuah Afoakwah

**Affiliations:** ^1^ Department of Food Science and Technology University for Development Studies Tamale Ghana

**Keywords:** beany‐flavor, functional properties, okara, phytochemicals, spontaneous fermentation, thermal pretreatment

## Abstract

Okara is a nutrient‐rich soybean‐processing byproduct whose food use is limited by undesirable beany‐flavor and variable functional properties. This study examined the individual and interactive effects of thermal pretreatment and the duration of spontaneous fermentation on the sensory, physicochemical, compositional, phytochemical, and functional characteristics of okara flour. Wet okara was either left untreated or heated at 90°C–100°C for 5 min, then spontaneously fermented at 25°C ± 3°C for 0, 12, 24, 36, or 48 h in a randomized 2 × 5 factorial design with three independent processing replicates. Data were analyzed by blocked two‐factor analysis of variance followed by Tukey's HSD test. Fermentation duration affected beany‐flavor intensity (*p* < 0.001), whereas thermal pretreatment and the interaction were not significant. Thermally pretreated okara fermented for 12 h recorded the lowest beany‐flavor intensity (2.24 ± 1.10), while scores exceeded 6.6 after 24–48 h. Sensory color and texture were not significantly affected (*p* > 0.05). Significant thermal pretreatment × fermentation duration interactions occurred for pH, titratable acidity, and water absorption capacity (*p* < 0.001). Crude protein ranged from 24.54 to 29.84 g/100 g and was affected by thermal pretreatment (*p* = 0.049) and fermentation duration (*p* < 0.001). Fermentation duration also affected total phenolics, phytate, and tannins (*p* < 0.001), whereas Hausner ratio and Carr's index remained unchanged. Among the treatment combinations evaluated, the thermally pretreated 12‐h sample had the lowest observed beany‐flavor intensity while retaining the broader compositional and functional characteristics assessed. This treatment‐level observation should be interpreted descriptively because the thermal pretreatment × fermentation‐duration interaction for beany‐flavor intensity was not significant.

## Graphical abstract

Thermal pretreatment and spontaneous fermentation were evaluated for their effects on the quality characteristics of okara flour. Fermentation duration significantly affected beany‐flavor intensity, with thermally pretreated okara fermented for 12 h showing the lowest observed intensity (2.24 ± 1.10). The graphical abstract summarizes the processing sequence and the principal quality responses evaluated.

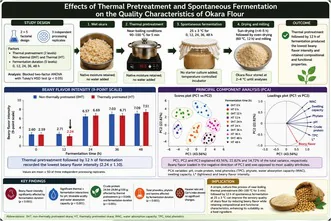

## 1 Introduction

Okara, the insoluble residue generated during soymilk and tofu production, is a nutritionally valuable soybean‐processing by‐product with potential for food valorization. Its composition varies with soybean variety and processing conditions, but dried okara has been reported to contain approximately 50% dietary fiber, 25% protein, and 10% lipid, together with other nutritional and bioactive constituents (Li et al. 2012; Feng et al. 2021). Despite this composition, substantial quantities remain underutilized or are directed to low‐value uses such as animal feed and fertilizer (Li et al. 2012; Feng et al. 2021). Converting okara into a suitable food ingredient could increase the value recovered from soybean processing.

Food utilization of okara is constrained by its high perishability in the fresh state and sensory characteristics such as undesirable beany/off‐odors and a fibrous or gritty mouthfeel (Feng et al. 2021; Vong and Liu 2017). Fermentation can modify these characteristics through microbial and enzymatic transformations. Solid‐state fermentation with *Bacillus subtilis* WX‐17 increased the levels of total amino acids and fatty acids and enhanced antioxidant activity relative to unfermented okara (Mok et al. 2019). Yeast fermentation has also reduced major off‐odor compounds, including hexanal and trans‐2‐hexenal, while generating additional aroma‐active compounds (Vong and Liu 2017). Recent evidence continues to identify microbial fermentation as a promising strategy for improving the nutritional and functional utilization of okara (Tuly and Ma 2024).

The thermal history of soybean curd residue may also influence subsequent fermentation. Oh et al. (2004) observed differences in acidification and viable microbial populations during fermentation of residues from hot‐ and cold‐processing systems. Lee et al. (2005) reported greater peptide and fibrinolytic‐enzyme production during *Bacillus firmus* NA‐1 fermentation of heat‐treated soybean curd residue than of the corresponding non‐heat‐treated material. Mok et al. (2020) subsequently used heat‐treated okara for *B. subtilis* WX‐17 fermentation and reported changes in metabolites, phenolic content, and antioxidant activity. However, because their fermentation experiment lacked an equivalent non‐heat‐treated fermented treatment, the specific effect of thermal pretreatment could not be separated from the subsequent fermentation response.

Previous investigations therefore suggest that thermal processing can influence subsequent fermentation, but an important methodological gap remains. Most studies have used selected starter cultures and focused on microbial growth, enzyme or peptide production, metabolite formation, or antioxidant characteristics (Oh et al. 2004; Lee et al. 2005; Mok et al. 2019, 2020). Less is known about how a defined short‐duration thermal pretreatment alters okara's response to spontaneous fermentation over time when both factors are evaluated in the same factorial experiment. A factorial approach permits separate estimation of thermal pretreatment, fermentation duration, and their interaction across sensory, physicochemical, compositional, phytochemical, and functional characteristics.

This study investigated the individual and interactive effects of thermal pretreatment and the duration of spontaneous fermentation on the physicochemical, proximate, phytochemical, functional, flowability, and sensory characteristics of okara flour. Particular attention was paid to beany‐flavor intensity, in response to concerns identified in the preceding consumer survey (Bawa et al. 2026), while color, texture, protein content, phenolic and antinutritional components, and hydration‐related properties were also evaluated. It was hypothesized that (i) thermal pretreatment and fermentation duration would independently alter selected properties of okara flour and that (ii) thermal pretreatment would modify responses to fermentation, producing thermal pretreatment × fermentation duration interactions for some measured characteristics.

## 2 Materials and Methods

### 2.1 Procurement of Okara Samples

Fresh wet okara was obtained from two artisanal tofu and soymilk processors near the University for Development Studies (UDS), Nyankpala Campus, Tolon District, Northern Region, Ghana. At each collection and processing occasion, okara was obtained from both processors, transferred into food‐grade polyethylene bags, and transported to the laboratory within approximately 15 min of collection, where it was held at 4°C until processing. The okara samples obtained from the two processors were combined and thoroughly mixed to form a homogeneous bulk sample before allocation to the experimental treatments. The study comprised three independent processing replicates. Because only five samples could be processed at a time, each replicate was completed over two collection and processing occasions, yielding six collection occasions in total.

#### 2.1.1 Experimental Design and Processing of Wet Okara Into Flour

The experiment used a randomized 2 × 5 factorial design with two thermal pretreatment conditions (non‐thermally pretreated and thermally pretreated) and five spontaneous fermentation durations (0, 12, 24, 36, and 48 h). Each of the 10 treatment combinations was prepared once within each of three independent processing replicates, yielding 30 experimental samples. Owing to processing‐capacity limitations, five treatment combinations were prepared per collection and processing occasion; consequently, each complete replicate was conducted over two occasions. Within each replicate, the order in which the 10 treatment combinations were prepared was randomized, with five samples prepared on each occasion.

For thermal pretreatment, wet okara was transferred to a covered stainless‐steel pot and heated at 90°C–100°C for 5 min. Temperature was monitored with a digital K‐type thermocouple thermometer (HI935005, Hanna Instruments, Spain) fitted with a metallic probe, and the heat source was adjusted to maintain the target range. No water was added during heating. This treatment was selected to approximate the near‐boiling heat exposure associated with traditional hot processing during okara production (O'Toole 1999). After heating, the okara was cooled to room temperature (25°C–28°C), thoroughly mixed in stainless‐steel basins, transferred to covered 4‐L food‐grade containers, and spontaneously fermented in a temperature‐controlled incubator (Medilow J.P. Selecta, S.A., 2101270, Spain) at 25°C ± 3°C for 0, 12, 24, 36, or 48 h. Corresponding non‐thermally pretreated samples underwent the same fermentation procedure without prior heating. No starter culture was added, and the naturally occurring microbial population responsible for fermentation was not characterized.

At the end of each fermentation period, samples were spread in thin layers on clean black polyethylene sheets and sun‐dried for approximately 4–5 h. The temperature measured during drying was approximately 35°C–38°C, consistent with the open‐air drying approach used in traditional processing of fermented cereal flours in northern Ghana (Atuna et al. 2022). Because drying was conducted under open‐air conditions, solar radiation, relative humidity, and air movement were not controlled and could vary during the drying period; the reported temperature range therefore represents the observed drying conditions rather than a controlled drying temperature.

### 2.2 Laboratory Analysis of Dried Okara Samples

#### 2.2.1 Sensory Analysis

Sensory evaluation of the pretreated okara flour was conducted in a controlled sensory laboratory with individual booths under environmental conditions consistent with ISO 8589:2007 (ISO 2007). The laboratory was maintained under uniform lighting, in an odor‐free environment, and at an ambient temperature of 22°C ± 2°C. Twenty‐five final‐year food science students served as semi‐trained assessors. Before evaluation, they were oriented and familiarized with the procedure, sensory attributes, intensity scales, and reference materials for beany flavor, color, and texture, following principles described in ISO 8586:2012 (ISO 2012). For beany flavor, graded liquid reference solutions representing increasing intensities, including a zero‐intensity reference, were used during familiarization.

The sensory samples comprised the 10 thermal pretreatment × fermentation duration combinations from each of the three independent processing replicates, for a total of 30 samples. Each assessor evaluated all 30 samples across the 3‐day evaluation period. The sensory presentation therefore followed a randomized complete‐block arrangement, with assessor serving as the presentation block. Approximately 10 g of each flour sample was presented in identical transparent, lidded 50‐mL disposable cups coded with randomized three‐character identifiers. Beany flavor, color, and texture were evaluated using 9‐point structured intensity scales with attribute‐specific anchors, following general sensory scaling principles described by Lawless and Heymann (2010). The scales measured attribute intensity rather than hedonic liking or consumer acceptability.

To limit sensory fatigue and carryover, evaluation was conducted over 3 days. Samples from the three independent processing replicates were randomly allocated across sessions, with each assessor evaluating up to 10 samples per day in two sessions of five, separated by a 5‐min break. Presentation order within each session was randomized. Responses were recorded using a structured Google Form aligned with the sensory scales, with paper forms retained as backup, and checked for completeness and consistency before statistical analysis.

Assessor ratings for each sample were summarized within the corresponding processing replicate, and the resulting replicate‐level treatment means were subjected to the factorial analysis described in Section 2.3. All participants provided informed consent. Ethical approval was granted by the Institutional Review Board of the University for Development Studies, Ghana (UDS/RB/0177/25).

#### 2.2.2 Physicochemical Analysis

##### 2.2.2.1 Physical Analysis

The pH of the okara flour samples was measured using a calibrated pH meter (Basic 20, Crison, Spain). The instrument was calibrated with certified buffers at pH 4.00 and 7.00. Five grams of flour was dispersed in 20 mL of distilled water (1:4, w/v), stirred for 5 min, allowed to stand for 10 min, and the pH of the resulting suspension was measured.

Titratable acidity (TTA) was determined by homogenizing 10 g of dried okara flour with 90 mL of distilled water. A 25‐mL aliquot was titrated with 0.1 M NaOH to pH 8.2, and TTA was expressed as the volume (mL) of 0.1 M NaOH required to reach the endpoint (Wolter et al. 2014).

Instrumental color was measured using a CR‐400 Chroma Meter (Konica Minolta, Japan) in the CIE L*a*b* color space. The instrument was calibrated against a standard white tile before measurement. Three measurements were taken at randomly selected positions on the flour surface. L* represents lightness, a* the red–green coordinate, and b* the yellow–blue coordinate (CIE 2004). Whiteness index (WI) and chroma (C*) were calculated as follows:

(1) $$W_{1}=100-\sqrt{\left({\left(100-L^{*}\right)}^{2}+{\left(a^{*}\right)}^{2}+{\left(b^{*}\right)}^{2}\right)}:$$

(2) $$C^{*}=\sqrt{\left({\left(a^{*}\right)}^{2}+{\left(b^{*}\right)}^{2}\right)}:$$

##### 2.2.2.2 Chemical Analysis

The proximate composition of the dried okara flours was determined using standard analytical procedures in accordance with AOAC methods (Latimer Jr. 2023). Moisture content was determined gravimetrically by oven drying. Approximately 5 g of flour was weighed into a clean, pre‐dried, and pre‐weighed moisture can and dried in a hot‐air oven (Thermo Scientific Heratherm, Germany) at 105°C until a constant mass was reached. The dried samples were cooled before reweighing, and moisture content was calculated from the loss in mass during drying as:

(3) $$\text{Moistuer}\;\left(\frac{g}{100g}\right)=\frac{W_{1}-W_{2}}{W_{s}}\times 100:$$

where *W*_1_ is the mass of the moisture can plus sample before drying, *W*_2_ is the mass of the moisture can plus sample after drying, and *W*_*s*_ is the initial mass of the sample.

Crude protein was determined by the Kjeldahl method using a block digester (DK 6 VELP‐F30100182, Italy) and a nitrogen distiller (j. Pro‐Nitro II, Spain). Total nitrogen was measured after digestion and distillation and converted to crude protein using a nitrogen‐to‐protein conversion factor of 6.25:

(4) $$\text{Crude protein}\;\left(\frac{g}{100g}\right)=\text{Total nitrogen}\;\left(\%\right)\times 6.25:$$

Crude fat was determined gravimetrically using a Soxtec extraction system (Det‐Grass6plazas‐4000842, Spain). Approximately 3 g of flour was weighed into an extraction thimble, placed in the extraction cartridge, and mounted in the Soxtec unit. Petroleum ether was used as the extraction solvent, and extracted lipid was collected in pre‐weighed aluminum cups. After extraction and solvent removal, the cups containing the recovered lipid were weighed, and crude fat content was calculated as follows:

(5) $$\text{Crude}\;\mathrm{fat}\;\left(\frac{g}{100g}\right)=\frac{W_{f}-W_{0}}{W_{s}}\times 100:$$

where *W*_*f*_ is the mass of the extraction cup plus extracted lipid, *W*_0_ is the mass of the empty extraction cup, and *W*_*s*_ is the initial mass of the flour sample.

Ash content was determined gravimetrically by incineration. Clean porcelain crucibles were heated, cooled, and weighed before use. Approximately 3 g of flour was weighed into each crucible and ashed in a muffle furnace (SELECT‐HORN‐200367, Spain) at 550°C. After ashing, the crucibles containing the residual ash were cooled in a desiccator and weighed. Ash content was calculated as:

(6) $$\mathrm{Ash}\left(\frac{g}{100g}\right)=\frac{W_{a}-W_{0}}{W_{s}}\times 100:$$

where *W*_*a*_ is the mass of the crucible plus ash, *W*_0_ is the mass of the empty crucible, and *W*_*s*_ is the initial mass of the sample.

Total carbohydrate was calculated by difference as follows:

(7) $$\text{Total Carbohydrate}\left(\frac{g}{100g}\right)=100-\left(\text{moisture}+\text{Crude protein}+\text{Crude}\;\mathrm{fat}+\mathrm{ash}\right):$$

Estimated energy value was calculated from crude protein, crude fat, and carbohydrate using Atwater conversion factors of 4, 9, and 4 kcal/g, respectively:

(8) $$\text{Energy}\;\left(\frac{\text{kcal}}{100g}\right)=\left(4\times \text{protein}\right)+\left(9\times \mathrm{fat}\right)+\left(4\times \text{carbohydrate}\right):$$

All proximate composition results were expressed as g/100 g of flour, except energy, which was expressed as kcal/100 g.

#### 2.2.3 Phytochemical Analysis

##### 2.2.3.1 Total Phenolic Content

Total phenolic content (TPC) was determined using the spectrophotometric method described by Bezuneh and Kebede (2015), with modifications. Phenolic compounds were extracted from 1.0 g of dried okara flour using 45 mL of acidified 70% acetone (acetone:water:acid, 70:29.5:0.5, v/v/v), with the extraction solvent prepared according to Xu and Chang (2007). The mixture was thoroughly mixed and filtered through Whatman No. 1 filter paper, and the resulting extract was used for TPC determination. The reaction was developed using ferric chloride (FeCl₃) and 1, 10‐phenanthroline under the specified assay conditions, and absorbance was measured at 511 nm using a BIOBASE UV/Vis spectrophotometer (BK‐V1000, China). Gallic acid was used to construct the calibration curve, and TPC was expressed as μg gallic acid equivalents per gram of dry sample (μg GAE/g).

##### 2.2.3.2 Phytate Content

Phytate content was determined according to Makkar et al. (2007). Approximately 0.7 g of dried okara flour was extracted with 50 mL of 3% trichloroacetic acid (TCA) in a water bath with integrated shaking at 100 rpm for 30 min. After centrifugation at 3000 rpm for 10 min, an aliquot of the supernatant was reacted with FeCl₃ to precipitate phytate as ferric phytate. The precipitate was washed, treated with NaOH, and dissolved in hot HNO₃ according to the referenced procedure. The resulting solution was made to a known volume, and absorbance was measured at 480 nm using a BIOBASE spectrophotometer (BK‐V1000, China) against a Fe (NO₃)₃ calibration curve. Phytate‐phosphorus was calculated from measured iron content, assuming a 4Fe:6P molar ratio in the precipitate, as described by Makkar et al. (2007) and phytate content was expressed as mg/100 g of flour.

(9) $$\left[\frac{\left(\text{Phytate}-P\right)}{100}\right]\mathrm{mg}/100g\;\text{sample}=\frac{\left[\mathrm{Fe}\;\left(\mathrm{\mu g}\right)\times 15\;\right]}{\text{Sample weight}\;\left(g\right)}:$$

##### 2.2.3.3 Tannin Content

Tannin content was determined using the polyvinylpolypyrrolidone (PVPP)‐binding method described by Makkar et al. (2007). One gram of dried okara flour was extracted with 10 mL of 70% aqueous acetone and agitated in a water bath with integrated shaking (Unitronic OR, Spain) for 20 min at room temperature. Extracts were cooled on ice for 25 min and centrifuged at 3000 rpm for 10 min. Total phenolics were determined in an aliquot of the supernatant using the Folin–Ciocalteu method. A second aliquot was treated with PVPP to bind tannin phenolics, and residual non‐tannin phenolics were determined similarly. Absorbance was measured at 725 nm against a reagent blank using a BIOBASE spectrophotometer (BK‐V1000, China), and concentrations were obtained from a tannic acid calibration curve. Tannin content was expressed as grams of tannic acid equivalents per 100 g of flour (g TAE/100 g) and calculated as follows:

(10) $$\text{Total phenolics}\;\left(\frac{g}{100g}\right)-\text{Non tannin phenolics}\;\left(\frac{g}{100g}\right)=\text{Tannins}\;\left(\frac{g}{100g}\right):$$

#### 2.2.4 Functional and Flowability Analysis

##### 2.2.4.1 Water and Oil Absorption Capacities (WAC and OAC)

Water and oil absorption capacities were determined using a modification of Beuchat (1977). For water absorption capacity (WAC), 1.0 g of okara flour was weighed into a pre‐weighed centrifuge tube, mixed thoroughly with 10 mL of distilled water, allowed to stand for 30 min at room temperature (approximately 28°C), and centrifuged at 3000 rpm for 15 min. The supernatant was carefully decanted, and the tube containing the hydrated sediment was weighed. Oil absorption capacity (OAC) was determined under the same conditions using 10 mL of sunflower oil instead of water. WAC and OAC were calculated as the mass of liquid retained per gram of dry flour:

(11) $$\text{Absorption capacity}\left(\frac{g}{g}\right)=\frac{\left(W_{f}-W_{0}\right)-W_{s}}{W_{s}}:$$

*W*_0_ = weight of the empty centrifuge tube, *Ws* = initial weight of dry flour, and *W*_*f*_ = weight of the centrifuge tube plus the hydrated or oil‐bound sediment after decanting. WAC and OAC were expressed as g water/g dry flour and g oil/g dry flour, respectively.

##### 2.2.4.2 Swelling Power (SP)

Swelling power was determined using a modification of Elkhalifa and Bernhardt (2013). A 5 g of okara flour was dispersed in 30 mL of distilled water in a pre‐weighed 50 mL centrifuge tube and heated in a water bath at 100°C for 30 min. Samples were cooled to room temperature and centrifuged at 3000 rpm for 15 min., the supernatant was carefully decanted, and the swollen sediment was weighed. Swelling power was calculated as the mass of wet sediment relative to the initial dry flour mass and expressed as g/g.

(12) $$\mathrm{SP}\left(\frac{g}{g}\right)=\frac{\text{mass of}\;\mathrm{wet}\;\text{sediment}\;\left(g\right)}{\text{initial mass of}\;\mathrm{dry}\;\text{flour}\;\left(g\right)}:$$

##### 2.2.4.3 Bulk and Tapped Bulk Densities

Bulk and tapped bulk densities were determined using a method adapted from Ashogbon and Akintayo (2012). A clean, dry graduated cylinder was weighed, and dried okara flour was introduced without compaction to a volume of 15 mL. The cylinder was reweighed and the flour mass obtained by difference. The initial flour volume was recorded as the bulk volume. The cylinder was then tapped 60 times, or until no further reduction in sample volume occurred, and the final volume was recorded as the tapped volume. Bulk and tapped bulk densities were calculated as follows:

(13) $$\rho_{\text{bulk}}=\frac{m}{V_{\text{bulk}}}:$$

(14) $$\rho_{\text{tapped}}=\frac{m}{V_{\text{tapped}}}:$$

where m is the mass of flour (g), *V*_bulk_ is the untapped flour volume (cm^3^), and *V*_tapped_ is the volume after tapping (cm^3^). Results were expressed as g/cm^3^.

##### 2.2.4.4 Carr's Compressibility Index (CI) and Hausner Ratio (HR)

The flowability indices were derived from the analytical values of bulk and tapped bulk density values using the following equations:

(15) $${\text{Carr}}^{’}s\;\mathrm{CI}\;\left(\%\right)=\left(\frac{\rho_{\text{taped}}-\rho_{\text{Bulk}}}{\rho_{\text{tapped}}}\right)\times 100:$$

(16) $$\mathrm{HR}=\frac{\rho_{\text{tapped}}}{\rho_{\text{Bulk}}}:$$

### 2.3 Statistical Analysis

Data from physicochemical, proximate, phytochemical, color, functional, and flowability analyses were expressed as mean ± standard deviation of three independent processing replicates. The effects of thermal pretreatment and fermentation duration were evaluated using two‐factor analysis of variance (ANOVA), with processing replicate included as a blocking factor. The statistical model was:

(17) $$Y_{\mathrm{ijk}}=\mu +R_{k}+H_{i}+F_{j}+{\left(H\times F\right)}_{\mathrm{ij}}+\varepsilon_{\mathrm{ijk}}:$$

where *Y*_*ijk*_ is the observed response, *μ* is the overall mean, *R*_k_ is the effect of processing replicate, *H*_*i*_ is the effect of thermal pretreatment (0HT and HT), *F*_*j*_ is the effect of fermentation duration (0, 12, 24, 36, and 48 h), (*H* × F)_*ij*_ is the interaction between thermal pretreatment and fermentation duration, and εijk is the residual error. Statistical significance was set at *p* < 0.05. Where appropriate, differences among treatment‐combination means were evaluated using Tukey's honestly significant difference (HSD) test.

For sensory evaluation, assessor ratings of beany‐flavor, color, and texture were summarized for each treatment within each independent processing replicate. Although assessor served as the block for sample presentation, inference on processing effects was based on the three independent processing replicates. Replicate‐level treatment means were therefore analyzed according to the 2 × 5 factorial structure, with processing replicate included as a blocking factor, to estimate the main effects of thermal pretreatment and fermentation duration and their interaction. Tukey's HSD test was applied where appropriate at *p* < 0.05.

Principal component analysis (PCA) was used as an exploratory multivariate analysis to examine relationships among selected quality attributes. Treatment means for pH, crude protein, total phenolic content, phytate, water absorption capacity, swelling power, instrumental lightness (L*), and beany‐flavor intensity were standardized to zero mean and unit variance before analysis. PCA was conducted on the 10 thermal pretreatment × fermentation duration treatment profiles and interpreted descriptively, given the small number of treatment profiles rather than as an inferential test of treatment differences.

Statistical analyses were conducted in Python version 3.13.5. Factorial ANOVA and related procedures were performed using statsmodels version 0.14.6 (Seabold and Perktold 2010), while PCA was conducted using scikit‐learn version 1.8.0 (Pedregosa et al. 2011).

## 3 Results and Discussion

### 3.1 Sensory Attributes of Pretreated Okara Flour

Fermentation duration was the primary factor shaping the sensory profile of okara flour, with the strongest response observed for beany‐flavor intensity (Table 1). Fermentation duration had a pronounced effect on beany‐flavor (*F* = 197.887, *p* < 0.001, partial *η* ^2^ = 0.978), whereas thermal pretreatment (*F* = 0.120, *p* = 0.733) and the thermal pretreatment × fermentation duration interaction (*F* = 1.036, *p* = 0.416) were not significant. Processing replicate was also insignificant (*p* = 0.673). The absence of an interaction indicates that changes in beany‐flavor across fermentation durations followed broadly similar patterns in thermally and non‐thermally pretreated okara.

**TABLE 1:** Effects of thermal pretreatment and fermentation duration on sensory intensity scores of okara flour.

Processing condition		Sensory intensity score, mean ± SD		
Thermal pretreatment	Fermentation duration (h)	Beany‐flavor	Color	Texture
0HT	0	2.600 ± 1.151	3.653 ± 1.502	3.707 ± 1.468
0HT	12	2.707 ± 1.450	3.373 ± 1.333	4.013 ± 1.447
0HT	24	6.573 ± 1.463	4.373 ± 1.088	4.853 ± 1.591
0HT	36	7.027 ± 1.355	4.893 ± 1.226	4.573 ± 1.612
0HT	48	7.093 ± 1.265	3.387 ± 1.335	4.320 ± 1.645
HT	0	2.587 ± 1.326	2.827 ± 1.032	4.027 ± 1.551
HT	12	2.240 ± 1.101	3.467 ± 1.687	4.773 ± 1.448
HT	24	6.693 ± 1.602	3.920 ± 1.505	4.173 ± 1.631
HT	36	6.707 ± 1.523	4.253 ± 1.462	4.173 ± 1.359
HT	48	7.507 ± 1.223	4.413 ± 1.079	3.867 ± 1.277
Factorial ANOVA, *p* values				
Thermal pretreatment (H)		0.733	0.586	0.736
Fermentation duration (F)		< 0.001	0.057	0.788
H × F		0.416	0.294	0.412

*Note:* Values are the mean ± SD of sensory intensity scores from 25 assessors across three independent processing replicates. Each assessor evaluated all 30 samples across 3 days, with samples randomly allocated across sessions and presentation order randomized within sessions; this constituted a randomized complete‐block arrangement for sensory presentation. Assessor ratings were summarized within treatment and processing replicate, and factorial inference was based on the three independent processing replicates. Factorial *p* values were obtained from the blocked 2 × 5 ANOVA model. Because the thermal pretreatment × fermentation‐duration interaction was nonsignificant for all three sensory attributes, treatment‐combination superscript letters are not shown; interpretation is based on the factorial main effects. The sensory scales measured attribute intensity rather than hedonic liking or consumer acceptability.

Abbreviations: 0HT = non‐thermally pretreated okara, HT = thermally pretreated okara.

Beany‐flavor intensity remained low at 0 and 12 h but increased sharply from 24 h onward. In non‐thermally pretreated samples, scores increased from 2.60 ± 1.15 at 0 h and 2.71 ± 1.45 at 12 h to 6.57 ± 1.46, 7.03 ± 1.36, and 7.09 ± 1.26 at 24, 36, and 48 h, respectively. Thermally pretreated samples followed a comparable pattern, with scores of 2.59 ± 1.33 at 0 h and 2.24 ± 1.10 at 12 h increasing to 6.69 ± 1.60, 6.71 ± 1.52, and 7.51 ± 1.22 at 24, 36, and 48 h. The thermally pretreated 12‐h sample recorded the lowest observed score, but this treatment‐level minimum is descriptive because neither the thermal main effect nor the interaction was significant. The more robust finding was the marked increase in undesirable flavor beyond 12 h.

The early sensory response is consistent with fermentation‐induced modification of volatile compounds associated with soybean beany aroma. Wang et al. (2022) reported substantial reductions in beany‐flavor compounds during fungal fermentation of okara, including a marked decrease in hexanal and formation of new aroma‐active compounds. Keong et al. (2023) likewise found that fermentation with selected *Bacillus subtilis* and *Bacillus coagulans* strains reduced undesirable green and beany notes. Microbial conversion or degradation of aldehydes and other lipid‐derived volatiles can contribute to these changes (Tao et al. 2022).

The marked increase in beany‐flavor scale scores after 24 h shows that extending spontaneous fermentation did not produce progressive sensory improvement, but the higher scores should not be interpreted as evidence that soybean‐type beany flavor itself intensified. Although assessors were familiarized with graded beany‐flavor references, other pronounced fermentation‐derived flavor notes were not evaluated as separate attributes and may have contributed to ratings assigned on the beany‐flavor scale. Fermentation may reduce pre‐existing undesirable volatiles while simultaneously generating new aroma‐active metabolites. Keong et al. (2023) for example, detected increased acetoin, carboxylic acids, pyrazines, and branched‐chain fatty acids in *B. subtilis* ‐fermented okara, some associated with fermented, cheesy, or natto‐like notes. The elevated 24–48 h scores may therefore reflect, at least in part, accumulation of fermentation‐derived odorants rather than beany flavor alone. Because neither the microbial community nor volatile compounds were characterized, the relative contributions could not be determined.

Heating can influence beany‐flavor by suppressing lipoxygenase‐mediated formation of volatile oxidation products in soybean systems. Lv et al. (2011) showed that hot‐water treatment at 80°C–100°C progressively decreased lipoxygenase activity and several beany odor‐active compounds in soymilk, while lipoxygenase‐catalyzed oxidation of polyunsaturated fatty acids is an established route to aldehydes associated with soybean beany notes (Tao et al. 2022). In the present study, however, heating okara at 90°C–100°C for 5 min did not produce a significant overall reduction in beany‐flavor. Earlier soybean processing and volatile formation during subsequent spontaneous fermentation may have reduced the detectable contribution of the additional heating step. The lower numerical score of the thermally pretreated 12‐h sample should therefore not be interpreted as an independent thermal effect.

Perceived color was less responsive than flavor. Scores ranged from 2.83 ± 1.03 in the thermally pretreated 0‐h sample to 4.89 ± 1.23 in the non‐thermally pretreated 36‐h sample. Thermal pretreatment (*F* = 0.307, *p* = 0.586) and the interaction (*F* = 1.340, *p* = 0.294) were not significant, while fermentation duration approached but did not reach the 5% threshold (*F* = 2.809, *p* = 0.057, partial *η* ^2^ = 0.384). The numerical differences therefore do not support a systematic treatment effect on perceived color.

Texture varied even less across treatments, with mean intensity scores from 3.71 ± 1.47 to 4.85 ± 1.59. Thermal pretreatment (*F* = 0.117, *p* = 0.736), fermentation duration (*F* = 0.788, *p* = 0.548), and their interaction (*F* = 1.045, *p* = 0.412) were all nonsignificant. Processing therefore affected beany‐flavor much more strongly than perceived color or texture, and short‐duration fermentation associated with low beany‐flavor did not measurably worsen perceived coarseness.

Fermentation duration was therefore the dominant sensory factor. Beany‐flavor rose markedly after 12 h, whereas perceived color and texture remained comparatively stable. The thermally pretreated 12‐h sample had the lowest observed score (2.24 ± 1.10), but the nonsignificant interaction precludes a claim of thermal–fermentation synergy. Because the assessors rated attribute intensity rather than liking, lower beany‐flavor indicates a lower intensity rating for the measured attribute, not greater consumer acceptance.

### 3.2 Changes in pH and Titratable Acidity (TTA)

Thermal pretreatment, fermentation duration, and their interaction influenced the acid–base characteristics of okara flour (Figure 1). For pH, significant effects were observed for thermal pretreatment (*F* = 10.900, *p* = 0.004, partial *η* ^2^ = 0.377), fermentation duration (*F* = 317.276, *p* < 0.001, partial *η* ^2^ = 0.986), and their interaction (*F* = 10.081, *p* < 0.001, partial η^2^ = 0.691). TTA showed the same pattern, with significant effects of thermal pretreatment (*F* = 27.739, *p* < 0.001, partial *η* ^2^ = 0.606), fermentation duration (*F* = 102.043, *p* < 0.001, partial *η* ^2^ = 0.958), and the interaction (*F* = 47.168, *p* < 0.001, partial *η* ^2^ = 0.913). Processing replicate was nonsignificant for pH (*p* = 0.610) and TTA (*p* = 0.355).

**FIGURE 1:**
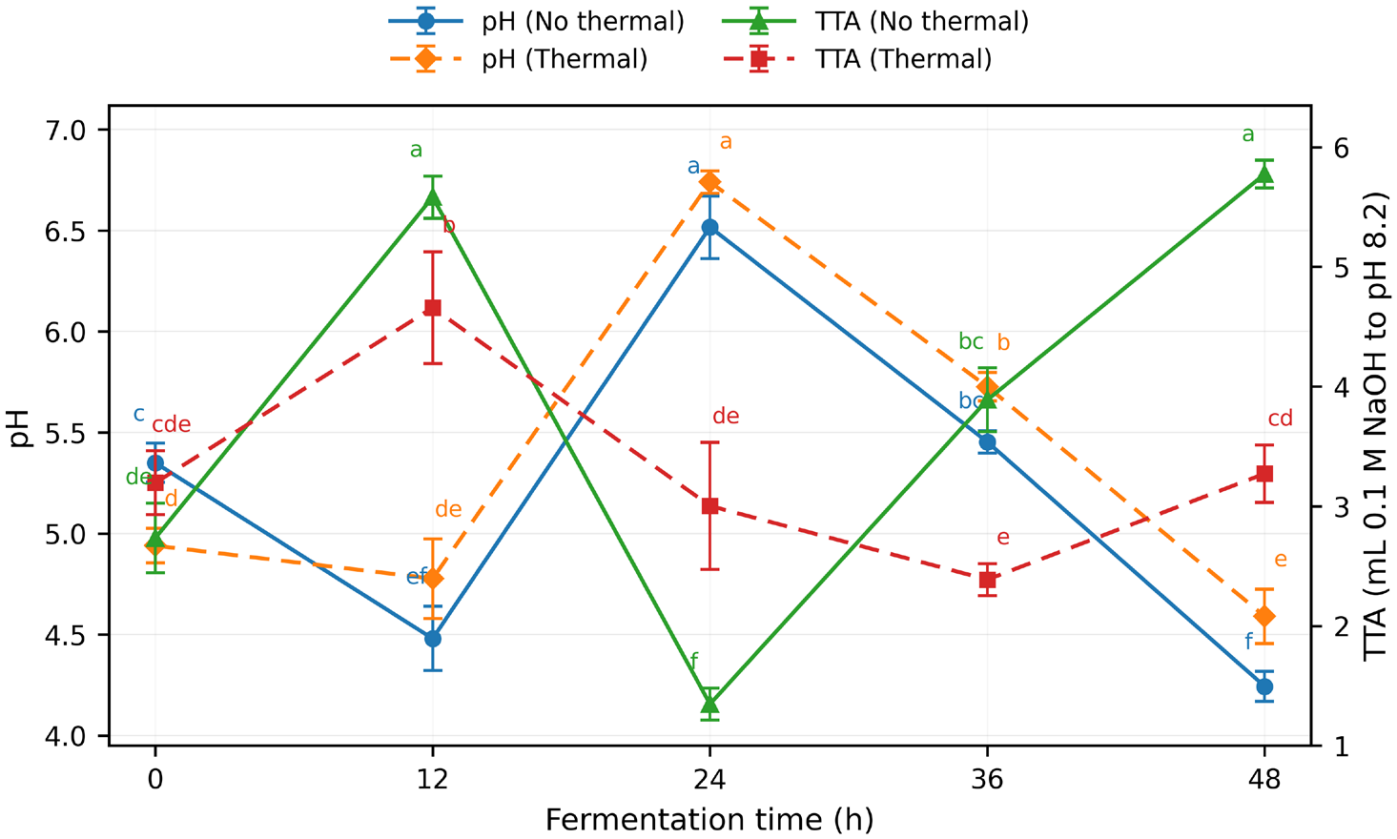
Effects of thermal pretreatment and fermentation duration on pH and titratable acidity of okara flour. Data points represent means of three independent processing replicates (*n* = 3); error bars represent standard deviations. HT = thermally pretreated okara, 0HT = non‐thermally pretreated okara, TTA = titratable acidity, expressed as the volume of 0.1 M NaOH required to reach pH 8.2. Different lowercase letters within each response variable indicate significant differences among treatment combinations according to Tukey's HSD test (*p* < 0.05).

The significant interactions indicate that pH and TTA responses during fermentation depended on thermal pretreatment. In non‐thermally pretreated samples, pH fell from 5.35 ± 0.10 at 0 h to 4.48 ± 0.16 at 12 h, rose to 6.52 ± 0.16 at 24 h, and declined to 4.24 ± 0.07 at 48 h. Thermally pretreated samples followed the same phasic pattern, from 4.94 ± 0.09 to 4.78 ± 0.20 and 6.74 ± 0.06 at 0, 12, and 24 h, before declining to 4.59 ± 0.14 at 48 h. Early acidification is consistent with naturally fermented systems containing okara (Rekha and Vijayalakshmi 2011). The 24‐h rebound indicates that acidification was not sustained throughout fermentation. Possible explanations include shifts in the dominant microbial populations, increased proteolysis, or the formation of alkaline nitrogenous metabolites during fermentation, as reported in spontaneous and legume fermentations (Allagheny et al. 1996; Atuna et al. 2025; Faisal et al. 2026). However, the microbial community, proteolytic activity, and fermentation metabolites were not determined in the present study; these mechanisms therefore remain possible explanations rather than established causes of the observed pH increase.

TTA also followed a treatment‐dependent trajectory. In non‐thermally pretreated okara, it rose from 2.73 ± 0.29 at 0 h to 5.58 ± 0.18 at 12 h, fell to 1.35 ± 0.13 at 24 h, and reached 5.77 ± 0.12 at 48 h. Thermally pretreated samples varied less, from 3.20 ± 0.27 at 0 h to 4.66 ± 0.47 at 12 h, then declined to 2.39 ± 0.13 at 36 h and ended at 3.27 ± 0.24 at 48 h. The interaction therefore indicates that pretreatment modified acidity development, although the data cannot separate effects on indigenous microbiota, substrate accessibility, or buffering (Sahin et al. 2024).

pH and TTA showed opposing changes at several stages but were not exact mirror images. pH measures hydrogen‐ion activity, whereas TTA reflects titratable acidity and the matrix buffering capacity. Proteins and other ionisable constituents of okara can influence this relationship. Rekha and Vijayalakshmi (2011) likewise reported comparable pH values but different titratable acidity in okara‐containing fermented batter, and attributed the difference to buffering by soluble proteins, amino acids, and other soybean constituents. pH and TTA are therefore complementary indicators of fermentation chemistry rather than consistently inverse measures.

These physicochemical changes contextualize the sensory response. At 12 h, both groups acidified while beany‐flavor remained low; the pH rebound from 24 h coincided with the sharp increase in beany‐flavor intensity. Given the protein‐rich nature of okara, the subsequent rise in pH may be consistent with a shift toward proteolytic or alkaline fermentation, during which protein degradation and the formation of alkaline metabolites, including ammonia, can increase pH in fermented legume substrates (Owusu‐Kwarteng et al. 2022). This temporal correspondence suggests concurrent changes in fermentation chemistry and perceived flavor, but pH and TTA alone cannot establish such a transition or identify the compounds responsible. Microbial profiling together with measurements of volatile compounds and nitrogenous metabolites would be required to determine the underlying mechanism. Importantly, microbiological safety was not verified in this study. Spontaneous fermentation was conducted without a starter culture, and the indigenous microbial population was not characterized. The pH of suspensions prepared from flour obtained after 24 h of fermentation increased to 6.52–6.74, indicating a marked reduction in acidity compared with the 12‐h treatments. Accordingly, the observed pH/TTA pattern should not be regarded as evidence of microbiological safety. Microbiological safety was not evaluated in the present study and should therefore be verified before practical food application of the processed okara flour.

### 3.3 Instrumental Color Attributes of Pretreated Okara Flour

Instrumental color was influenced primarily by fermentation duration rather than thermal pretreatment alone (Table 2). Thermal pretreatment had no significant main effect on L* (*p* = 0.514), a* (*p* = 0.179), b* (*p* = 0.146), chroma (*p* = 0.193), or whiteness index (*p* = 0.438). Fermentation duration significantly affected L* (*F* = 19.561, *p* < 0.001, partial *η* ^2^ = 0.813), a* (*F* = 7.959, *p* < 0.001, partial *η* ^2^ = 0.639), b* (*F* = 4.542, *p* = 0.010, partial *η* ^2^ = 0.502), chroma (*F* = 4.438, *p* = 0.011, partial *η* ^2^ = 0.497), and whiteness index (*F* = 6.444, *p* = 0.002, partial *η* ^2^ = 0.589). Significant thermal pretreatment × fermentation duration interactions occurred for a* (*p* = 0.034), b* (*p* = 0.005), chroma (*p* = 0.011), and whiteness index (*p* = 0.011), whereas the interaction for L* was not significant (*p* = 0.064).

**TABLE 2:** Instrumental color attributes of okara flour as influenced by thermal pretreatment and fermentation duration.

Thermal pretreatment	Fermentation duration (h)	Color parameters				
		L*	a*	b*	C*	Whiteness index
0HT	0	82.90 ± 0.99	2.83 ± 0.77^a^	20.54 ± 3.78^a^	20.63 ± 3.81^a^	73.09 ± 3.61^c^
0HT	12	81.49 ± 0.36	2.79 ± 0.05^a^	15.88 ± 0.33^b^	16.23 ± 0.23^b^	75.45 ± 0.48^abc^
0HT	24	81.13 ± 0.65	2.84 ± 0.10^a^	18.32 ± 0.32^ab^	18.51 ± 0.46^ab^	73.55 ± 0.62^abc^
0HT	36	81.28 ± 1.36	2.56 ± 0.30^ab^	17.18 ± 0.31^ab^	17.23 ± 0.13^ab^	74.45 ± 0.88^abc^
0HT	48	82.68 ± 0.83	1.81 ± 0.05^b^	15.77 ± 0.29^b^	15.74 ± 0.19^b^	76.50 ± 0.53^abc^
HT	0	83.78 ± 0.24	2.42 ± 0.13^ab^	15.77 ± 0.35^b^	16.07 ± 0.39^b^	77.24 ± 0.37^a^
HT	12	80.86 ± 0.30	3.03 ± 0.04^a^	16.95 ± 0.36^ab^	17.09 ± 0.13^ab^	74.25 ± 0.36^abc^
HT	24	80.16 ± 0.18	2.68 ± 0.03^a^	18.16 ± 0.31^ab^	18.29 ± 0.45^ab^	72.97 ± 0.31^c^
HT	36	80.24 ± 0.31	3.08 ± 0.07^a^	17.54 ± 0.20^ab^	17.79 ± 0.18^ab^	73.40 ± 0.33^bc^
HT	48	83.55 ± 0.70	2.35 ± 0.11^ab^	15.84 ± 0.19^b^	15.92 ± 0.56^b^	77.04 ± 0.60^ab^
Factorial ANOVA, *p* values						
Thermal pretreatment (H)		0.514	0.179	0.146	0.193	0.438
Fermentation duration (F)		< 0.001	< 0.001	0.010	0.011	0.002
H × F		0.064	0.034	0.005	0.011	0.011

*Note:* Values are mean ± SD of three independent processing replicates (*n* = 3). Factorial *p* values were obtained from the blocked 2 × 5 ANOVA model. Within columns showing a significant thermal pretreatment × fermentation‐duration interaction (a*, b*, C*, and whiteness index), means sharing at least one superscript letter are not significantly different according to Tukey's HSD test (*p* < 0.05). For L*, the interaction was nonsignificant; treatment‐combination superscript letters are therefore not shown, and interpretation is based on the significant fermentation‐duration main effect.

Abbreviations: 0HT = non‐thermally pretreated okara, a* = red–green coordinate, b* = yellow–blue coordinate, C* = chroma, F = fermentation duration, H = thermal pretreatment, HT = thermally pretreated okara, L* = lightness.

Lightness showed a non‐linear fermentation response. In thermally pretreated samples, L* decreased from 83.78 ± 0.24 at 0 h to 80.86 ± 0.30 at 12 h and 80.16 ± 0.18 at 24 h, remained low at 36 h, and recovered to 83.55 ± 0.70 at 48 h. Non‐thermally pretreated samples showed a smaller change, decreasing from 82.90 ± 0.99 at 0 h to 81.13 ± 0.65 at 24 h before increasing to 82.68 ± 0.83 at 48 h. Because the thermal main effect was nonsignificant, heating at 90°C–100°C for 5 min did not produce a consistent overall change in flour lightness. The intermediate reduction and subsequent recovery were primarily associated with fermentation duration.

Fermentation‐related color changes in okara are not necessarily unidirectional. Yue et al. (2023) similarly reported fermentation‐dependent changes in the color of okara‐derived insoluble dietary fiber, with L* increasing after lactic acid bacterial fermentation but decreasing after *K. marxianus* fermentation and co‐fermentation, alongside changes in a* and b* values. Although Yue et al. (2023) examined an isolated fiber fraction rather than whole flour, it shows that direction and magnitude depend on the fermentation system. The temporary reduction in lightness observed here may reflect changes in pigments, oxidation, or matrix structure, but these mechanisms were not measured and were not assigned to a specific pathway in this study.

More intensive heating can alter soybean color. Martínez et al. (2013) showed that heat‐treatment conditions affected soybean flour color during fluidized‐bed processing, while Kantrong et al. (2024) reported decreasing L* and b* values and increasing a* values with repeated extrusion treatment of okara powder under low‐moisture conditions. The shorter moist‐heat treatment used in the present study was milder and produced no consistent overall shift in L*, a*, b*, chroma, or whiteness index, indicating that the pretreatment did not cause pronounced instrumental discoloration.

Redness and yellowness were more strongly influenced by the combination of thermal pretreatment and fermentation duration. All a* values were positive but small. In non‐thermally pretreated samples, a* remained approximately 2.56–2.84 from 0 to 36 h before declining to 1.81 ± 0.05 at 48 h; thermally pretreated samples increased to 3.08 ± 0.07 at 36 h before declining to 2.35 ± 0.11 at 48 h. Positive b* values showed that yellowness remained the dominant chromatic component. The significant interactions for a* and b* indicate that thermal pretreatment altered the temporal pattern of color development rather than causing a consistent increase or decrease.

Chroma also showed an interaction pattern. Thermally pretreated samples increased from 16.07 ± 0.39 at 0 h to 18.29 ± 0.45 at 24 h, then declined to 15.92 ± 0.56 at 48 h. Non‐thermally pretreated samples declined from 20.63 ± 3.81 to 16.23 ± 0.23 at 12 h and ended at 15.74 ± 0.19 at 48 h. These fluctuations are consistent with treatment‐dependent color changes reported for fermented okara‐derived materials (Yue et al. 2023).

Whiteness index also varied with fermentation duration and its interaction with thermal pretreatment. In the thermally pretreated series, the whiteness index decreased from 77.24 ± 0.37 at 0 h to 72.97 ± 0.31 at 24 h, then recovered to 77.04 ± 0.60 at 48 h. Non‐thermally pretreated samples followed a more variable trajectory, from 73.09 ± 3.61 at 0 h to 76.50 ± 0.53 at 48 h. Recovery of both L* and whiteness index by 48 h in thermally pretreated samples indicates that the intermediate reduction in brightness was temporary rather than progressive darkening.

Instrumental and sensory color responses were not equivalent. Although fermentation duration significantly affected several instrumental parameters, perceived color intensity did not differ significantly with thermal pretreatment (*p* = 0.586), fermentation duration (*p* = 0.057), or their interaction (*p* = 0.294). Instrumentally detectable changes therefore did not translate into systematic differences perceived by the semi‐trained assessors.

Fermentation duration was the main determinant of instrumental color variation, while thermal pretreatment modified the temporal response of selected attributes without independently causing substantial discoloration. Changes were non‐linear and of modest absolute magnitude, with L* remaining above 80 across all treatments. The short thermal pretreatment therefore retained the relatively light appearance of the flour while fermentation produced transient shifts in brightness and chromatic characteristics.

### 3.4 Proximate Composition of Okara Flour

Proximate composition was influenced predominantly by fermentation duration, although some responses depended on thermal pretreatment (Figure 2). Processing replicate was nonsignificant for all proximate variables (*p* = 0.285–0.919). Fermentation duration significantly affected crude protein (*F* = 9.161, *p* < 0.001, partial *η* ^2^ = 0.671), ash (*F* = 11.365, *p* < 0.001, partial *η* ^2^ = 0.716), moisture (*F* = 5.775, *p* = 0.004, partial *η* ^2^ = 0.562), carbohydrate (*F* = 4.963, *p* = 0.007, partial *η* ^2^ = 0.524), crude fat (*F* = 6.089, *p* = 0.003, partial *η* ^2^ = 0.575), and energy value (*F* = 12.360, *p* < 0.001, partial *η* ^2^ = 0.733). Thermal pretreatment had significant main effects on protein (*p* = 0.049) and energy (*p* = 0.020), but not on ash, moisture, carbohydrate, or fat. Significant interactions occurred for ash (*p* = 0.040), fat (*p* = 0.001), and energy (*p* < 0.001), indicating treatment‐dependent fermentation responses.

**FIGURE 2:**
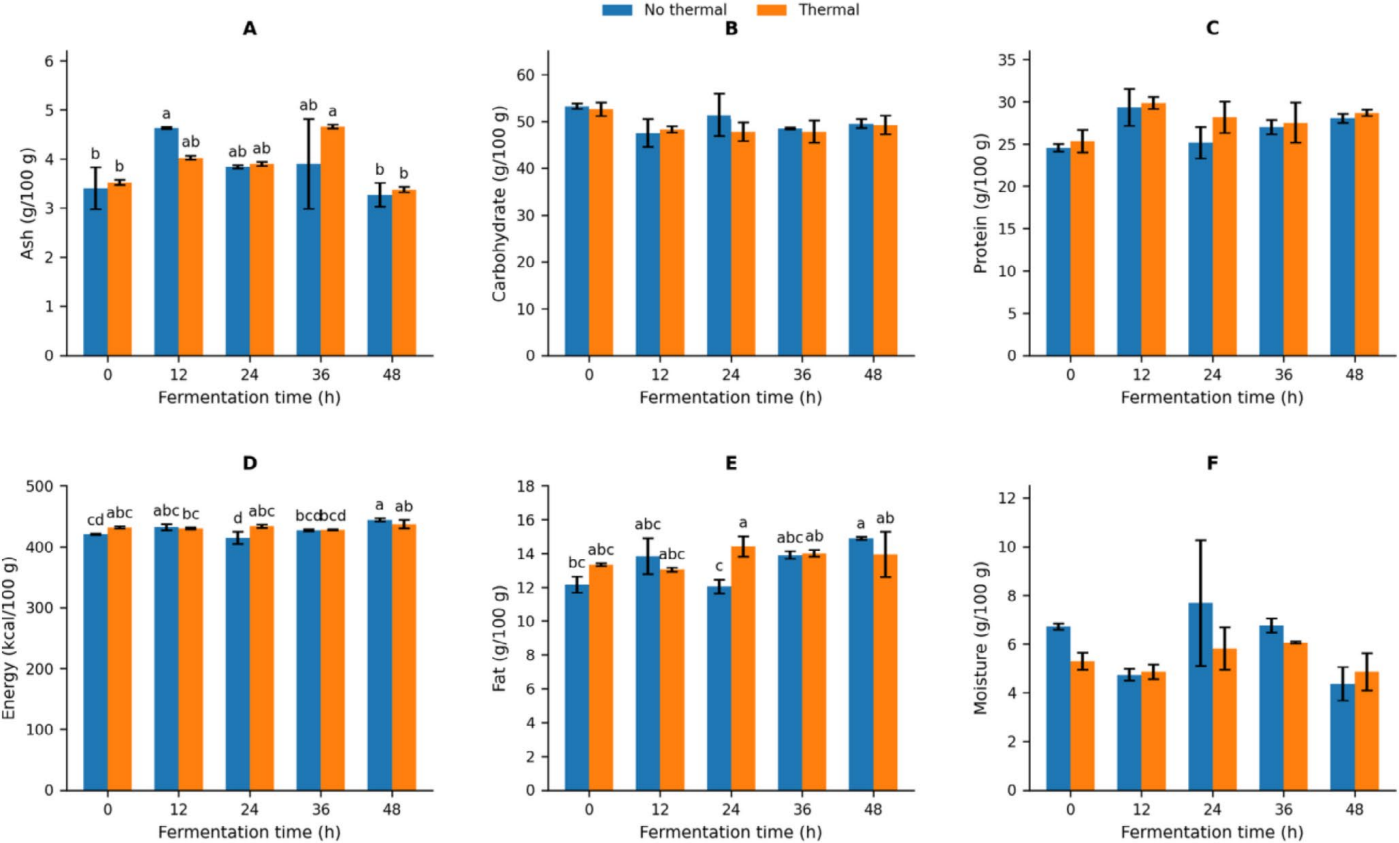
Effects of thermal pretreatment and fermentation duration on the proximate composition of okara flour. Bars represent means of three independent processing replicates (*n* = 3); error bars represent standard deviations. 0HT = non‐thermally pretreated okara; HT = thermally pretreated okara. Treatment‐combination letters are shown only for panels with a significant thermal pretreatment × fermentation‐duration interaction: (A) ash, (D) energy, and (E) fat. No treatment‐combination letters are shown for (B) carbohydrate, (C) protein, or (F) moisture because their interactions were nonsignificant; these responses are interpreted from the corresponding factorial main effects. Tukey's HSD test was applied at *p* < 0.05. Panels show: (A) ash, (B) carbohydrate, (C) protein, (D) energy, (E) fat, and (F) moisture.

Crude protein ranged from 24.54 ± 0.45 g/100 g in non‐thermally pretreated okara at 0 h to 29.84 ± 0.71 g/100 g in thermally pretreated okara at 12 h. Protein increased during the first 12 h in both groups and then remained approximately 25–29 g/100 g. The significant fermentation‐duration effect and nonsignificant interaction indicate a broadly similar temporal response with and without thermal pretreatment, while the smaller thermal main effect suggests an additional but more limited contribution of pretreatment.

Increases in measured protein during fermentation do not necessarily indicate net protein synthesis, as changes in other dry‐matter components and microbial metabolism can alter protein concentration proportionally. Fungal fermentation has increased the measured protein content of okara in some systems, although the magnitude of compositional changes varies with the microorganisms and fermentation conditions used (Sitanggang et al. 2020; Shi et al. 2020; Farniga et al. 2026). Fermentation can also promote proteolysis, increasing amino nitrogen, free amino acids, and smaller protein‐derived compounds (Sitanggang et al. 2020; Shi et al. 2020). The increase observed in the present study is therefore best interpreted as an increase in the measured protein proportion rather than evidence of new protein synthesis.

Ash content showed a significant fermentation‐duration effect and interaction, but no independent thermal effect. In non‐thermally pretreated okara, ash increased from 3.40 ± 0.43 g/100 g at 0 h to 4.63 ± 0.02 g/100 g at 12 h, then declined to 3.27 ± 0.24 g/100 g at 48 h. Thermally pretreated okara followed a different pattern, reaching 4.66 ± 0.04 g/100 g at 36 h, then declining at 48 h. The interaction therefore reflects differences in the timing of peak ash values rather than a consistent mineral‐increasing effect of heating.

Because ash represents the inorganic residue remaining after combustion, fermentation does not generate minerals. Changes in ash content per 100 g are better interpreted as proportional shifts driven by changes in the organic fraction of the dry matter; loss or utilization of organic substrates during fermentation can increase the relative concentration of mineral residue without raising the absolute mineral content. Fermentation may alter mineral chemical form or accessibility, but ash determination does not measure mineral bioaccessibility. Higher ash values should therefore not be taken directly as evidence of the “release of bound minerals.” Ash responses during okara fermentation are not uniform; for example, Shi et al. (2020) found no significant differences in ash among fresh and fermented okara treatments, whereas the broader composition of okara varies with soybean source and processing conditions (Asghar et al. 2023).

Total carbohydrate ranged from 47.47 ± 2.99 to 53.20 ± 0.54 g/100 g and was affected by fermentation duration, whereas thermal pretreatment and the interaction were nonsignificant. Both groups tended to show lower values after fermentation began, although the decline was not progressive across 48 h. Microbial utilization of fermentable carbohydrate provides a plausible explanation for the early decrease, as carbohydrate‐hydrolyzing activity has been reported in fermented soybean and okara systems (Li et al. 2022). Carbohydrate was calculated by difference; however, its value also reflects changes in protein, fat, ash, and moisture and cannot be interpreted solely as direct microbial consumption.

Moisture content remained low across treatments (4.36 ± 0.69–7.69 ± 2.58 g/100 g). Fermentation duration affected residual moisture, whereas thermal pretreatment (*p* = 0.074) and the interaction (*p* = 0.226) were nonsignificant. Because all treatments underwent drying after fermentation, final moisture differences may reflect treatment‐related variation in matrix structure, water binding, and equilibration during drying rather than the initial heat treatment. The comparatively large variation in non‐thermally pretreated okara at 24 h also warrants cautious interpretation. Low moisture alone does not ensure shelf stability; it also depends on water activity, microbial quality, packaging, and storage conditions.

Crude fat showed a significant thermal pretreatment × fermentation duration interaction (F = 7.073, *p* = 0.001, partial η^2^ = 0.611), despite a nonsignificant thermal main effect. In non‐thermally pretreated okara, fat varied from 12.15 ± 0.48 g/100 g at 0 h to 12.04 ± 0.41 g/100 g at 24 h, then increased to 14.89 ± 0.07 g/100 g at 48 h. Thermally pretreated samples ranged from 13.03 ± 0.12 to 14.41 ± 0.59 g/100 g and showed less evidence of a late increase. The interaction therefore indicates that pretreatment altered the temporal behavior of the measured lipid fraction rather than uniformly increasing or decreasing fat content.

As with protein, the higher crude fat percentage alone does not demonstrate lipid synthesis, particularly in the absence of lipidomic or mass‐balance evidence. Fermentation can disrupt cellular structures, alter lipid extractability, and change the proportional contribution of fat as other dry‐matter fractions are lost. Metabolomic analysis of fermented okara has shown organism‐dependent changes in lipid derivatives, including fatty acids, fatty acyls, glycerides, glycerophospholipids, and prenol lipids (Farniga et al. 2026). These findings provide context for the treatment‐dependent response but do not establish the mechanism underlying the changes observed in the present spontaneous fermentation.

Calculated energy ranged from 414.08 ± 10.19 kcal/100 g in non‐thermally pretreated okara at 24 h to 443.93 ± 2.46 kcal/100 g at 48 h and was affected by thermal pretreatment, fermentation duration, and their interaction. The interaction was most evident in the non‐thermally pretreated series, where the low value at 24 h was followed by the highest value at 48 h. Because energy was calculated from measured macronutrient fractions, these differences should be interpreted in light of the underlying composition; the high 48‐h value is particularly consistent with the higher measured crude‐fat content. A specific fermentation pathway cannot be inferred from calculated energy values.

Fermentation duration dominated proximate responses, while thermal pretreatment had selective effects, modifying ash and fat trajectories and contributing modestly to protein and calculated energy. Such variability is consistent with studies on okara showing dependence on the organism, fermentation mode, substrate preparation, and processing conditions (Asghar et al. 2023; Shi et al. 2020). The results indicate redistribution of measured nutrient fractions rather than generalized nutritional enrichment.

### 3.5 Phytochemical Composition of Okara Flour

Fermentation duration also exerted a stronger influence on measured phytochemical constituents than thermal pretreatment alone (Figure 3). Processing replicate was nonsignificant for total phenolic content, phytate, and tannins (*p* = 0.469–0.978). TPC was affected by fermentation duration (*F* = 12.414, *p* < 0.001, partial *η* ^2^ = 0.734) and the thermal pretreatment × fermentation duration interaction (*F* = 5.293, *p* = 0.005, partial *η* ^2^ = 0.540), but not by the thermal main effect (*p* = 0.647). Phytate was affected by fermentation duration (*F* = 8.840, *p* < 0.001, partial *η* ^2^ = 0.663), but not by thermal pretreatment (*p* = 0.569) or the interaction (*p* = 0.510). Tannin content showed effects of fermentation duration (*F* = 13.365, *p* < 0.001, partial *η* ^2^ = 0.748) and the interaction (*F* = 5.134, *p* = 0.006, partial *η* ^2^ = 0.533), while the thermal main effect was negligible (*p* = 0.982).

**FIGURE 3:**
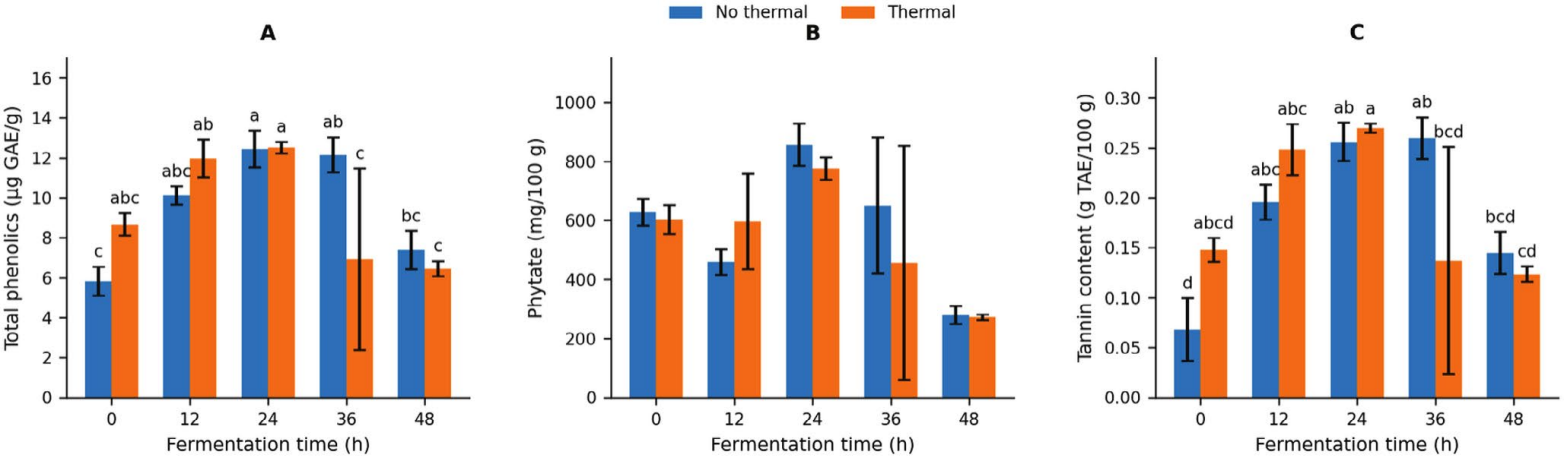
Effects of thermal pretreatment and fermentation duration on the phytochemical composition of okara flour. Bars represent means of three independent processing replicates (*n* = 3); error bars represent standard deviations. 0HT = non‐thermally pretreated okara; HT = thermally pretreated okara. Treatment‐combination letters are shown only for panels with a significant thermal pretreatment × fermentation‐duration interaction: (A) total phenolic content and (C) tannin content. No treatment‐combination letters are shown for (B) phytate because the interaction was nonsignificant; phytate is interpreted from the significant fermentation‐duration main effect. Tukey's HSD test was applied at *p* < 0.05.

TPC increased during early and intermediate fermentation in both treatment groups. In non‐thermally pretreated okara, values rose from 5.82 ± 0.73 μg GAE/g at 0 h to 10.12 ± 0.46 at 12 h and 12.44 ± 0.92 μg GAE/g at 24 h. Thermally pretreated samples increased from 8.66 ± 0.56 μg GAE/g at 0 h to 11.96 ± 0.95 and 12.51 ± 0.29 μg GAE/g at 12 and 24 h. Thereafter, responses diverged. TPC remained relatively high in the non‐thermally pretreated 36‐h sample but declined in the thermally pretreated series, with both groups reaching lower values at 48 h. This divergence explains the interaction and indicates that pretreatment modified the later trajectory of extractable phenolics rather than producing a uniform increase.

Increased measurable phenolics after fermentation have been reported for okara. Vong et al. (2018) observed a 197% increase in free phenolic acids following fungal–yeast coculture fermentation, while Mok et al. (2020) reported a 6.32‐fold increase in TPC after *Bacillus subtilis* fermentation. More recently, Farniga et al. (2026) observed 2.19‐ to 4.13‐fold increases in phenolic constituents across different fungal fermentations. Such increases may result in part from microbial and enzymatic modification of the okara matrix and the release of phenolics from conjugated or less‐extractable forms (Mok et al. 2020).

The increase observed through 24 h is consistent with greater extractability or assay availability but should not be interpreted as direct phenolic synthesis. Folin‐based measurement reflects the reducing capacity of the extracted fraction and is not entirely specific to phenolic compounds, as other reducing substances can contribute to the measured response (Sánchez‐Rangel et al. 2013). The subsequent decline may reflect further microbial transformation, oxidation, polymerization, or reduced extractability of phenolic constituents. Because individual phenolic compounds and relevant enzymes were not characterized, the data establish a temporal change in measurable TPC but do not identify a specific biochemical pathway.

Phytate concentrations ranged from 271.88 ± 9.48 mg/100 g in the thermally pretreated 48‐h sample to 855.57 ± 71.56 mg/100 g in the non‐thermally pretreated 24‐h sample. Both groups showed a non‐linear pattern, with relatively high values around 24 h followed by marked reductions at 48 h. Neither the main effect of thermal pretreatment nor its interaction was significant, indicating that thermal pretreatment at 90°C–100°C for 5 min did not measurably alter the overall phytate response. Fermentation duration accounted for the observed variation.

Phytate did not decline continuously; instead, it increased during intermediate fermentation before reaching its lowest concentration at 48 h. Defined fungal fermentation of okara has reduced the phytic acid content. Vong et al. (2018), for example, reported a 16% decrease after fermentation with *Rhizopus oligosporus* and *Yarrowia lipolytica*. More broadly, microbial or endogenous phytase activity can degrade phytate in cereal and legume substrates, although the extent depends on organism, pH, substrate, and fermentation conditions (Garrido‐Galand et al. 2021).

The transient increase may reflect changes in matrix accessibility and phytate recovery before appreciable degradation later in fermentation, while shifts in dry‐matter composition may also affect concentration per unit mass. These explanations remain tentative because phytase activity, inositol‐phosphate intermediates, and microbial succession were not measured. The decrease at 48 h is compatible with phytate degradation during extended fermentation but does not establish the mechanism. Nor should lower phytate be equated directly with improved mineral bioavailability because mineral speciation and bioaccessibility were not assessed (Sandberg 2002).

Tannin content showed a pronounced treatment‐dependent temporal response. In non‐thermally pretreated samples, the measured tannin fraction increased from 24 to 36 h, then declined at 48 h. Thermally pretreated samples also increased initially, peaking at 24 h, but declined earlier and more sharply thereafter. The nonsignificant main effect of thermal treatment, therefore, does not imply that pretreatment was irrelevant; the significant interaction indicates that it modified the temporal response of the measured tannin fraction.

The early increase in measurable tannins may reflect greater extraction of phenolic compounds initially associated with proteins, polysaccharides, or other matrix components. Fermentative hydrolysis can increase the recovery of soluble and free phenolics in okara and soybean systems (Vong et al. 2018; Zhang et al. 2022). Zhang et al. (2022), for example, observed changes in both soluble and insoluble phenolic fractions during fermentation, consistent with time‐dependent liberation and transformation of phenolic compounds. The subsequent decline in measurable tannins may reflect further transformation, oxidation, polymerization, or changes in extractability. The stronger decline after 24 h in thermally pretreated samples suggests that pretreatment altered this balance, although the present measurements cannot distinguish effects of substrate structure, endogenous enzymes, or the spontaneous microbial community.

Phytochemical responses were not uniformly favorable with longer fermentation. TPC increased through the short‐to‐intermediate interval, whereas phytate and tannins followed non‐linear trajectories. Because beany‐flavor remained low at 12 h but increased sharply thereafter, the data support short fermentation as a quality compromise, not a claim of uniform phytochemical optimization or improved mineral bioavailability.

### 3.6 Functional and Flowability Characteristics of Okara Flour

Thermal pretreatment and fermentation duration affected functional and flowability characteristics differently (Table 3). Bulk density was influenced by fermentation duration (*p* = 0.025), whereas thermal pretreatment (*p* = 0.690) and the interaction (*p* = 0.521) were nonsignificant. Tapped bulk density responded to thermal pretreatment (*p* = 0.003), fermentation duration (*p* < 0.001), and their interaction (*p* = 0.003). WAC was affected by fermentation duration (*p* < 0.001) and the interaction (*p* < 0.001), but not by the thermal main effect (*p* = 0.664). OAC varied with fermentation duration (*p* = 0.023), but not with thermal pretreatment (*p* = 0.484) or the interaction (*p* = 0.250). Swelling power was affected by thermal pretreatment (*p* < 0.001), fermentation duration (*p* < 0.001), and their interaction (*p* = 0.049). Hausner ratio and Carr's index were not significantly affected by either factor or their interaction (*p* > 0.05).

**TABLE 3:** Functional and flowability properties of okara flour as influenced by thermal pretreatment and fermentation duration.

Thermal pretreatment	Fermentation duration (h)	Bulk density (g/cm^3^)	Tapped bulk density (g/cm^3^)	WAC (g/g)	OAC (g/g)	Hausner ratio	Carr's index (%)	Swelling power (g/g)
0HT	0	0.375 ± 0.008	0.518 ± 0.015^e^	4.52 ± 0.57^abc^	2.98 ± 0.11	1.38 ± 0.07	27.45 ± 3.78	2.93 ± 0.06^bcd^
0HT	12	0.437 ± 0.034	0.613 ± 0.021^a^	3.75 ± 0.04^d^	3.70 ± 0.53	1.41 ± 0.09	28.77 ± 4.77	2.90 ± 0.10^cd^
0HT	24	0.401 ± 0.016	0.527 ± 0.000^de^	4.33 ± 0.13^bcd^	3.12 ± 0.17	1.32 ± 0.05	23.92 ± 3.05	2.80 ± 0.10^cd^
0HT	36	0.411 ± 0.009	0.579 ± 0.040^abc^	4.46 ± 0.36^abc^	3.45 ± 0.43	1.41 ± 0.08	28.78 ± 4.22	2.73 ± 0.06^d^
0HT	48	0.412 ± 0.020	0.589 ± 0.000^ab^	4.82 ± 0.03^ab^	3.71 ± 0.48	1.43 ± 0.07	30.03 ± 3.41	3.00 ± 0.10^abc^
HT	0	0.396 ± 0.019	0.527 ± 0.000^de^	4.65 ± 0.13^abc^	3.29 ± 0.13	1.33 ± 0.06	24.89 ± 3.52	3.17 ± 0.06^a^
HT	12	0.417 ± 0.017	0.567 ± 0.019^abcd^	4.16 ± 0.03^cd^	3.59 ± 0.07	1.36 ± 0.07	26.30 ± 3.99	2.87 ± 0.06^cd^
HT	24	0.395 ± 0.009	0.546 ± 0.017^bcde^	5.02 ± 0.20^a^	3.17 ± 0.04	1.38 ± 0.06	27.59 ± 3.40	2.90 ± 0.10^cd^
HT	36	0.412 ± 0.025	0.546 ± 0.017^bcde^	3.78 ± 0.02^d^	3.30 ± 0.05	1.33 ± 0.05	24.55 ± 2.62	2.97 ± 0.06^abc^
HT	48	0.401 ± 0.016	0.536 ± 0.017^cde^	4.09 ± 0.06^cd^	3.26 ± 0.03	1.34 ± 0.07	25.25 ± 3.86	3.13 ± 0.06^ab^
Factorial ANOVA, *p* values								
Thermal pretreatment (H)		0.690	0.003	0.664	0.484	0.112	0.128	< 0.001
Fermentation duration (F)		0.025	< 0.001	< 0.001	0.023	0.836	0.856	< 0.001
H × F		0.521	0.003	< 0.001	0.250	0.281	0.290	0.049

*Note:* Values are mean ± SD of three independent processing replicates (*n* = 3). Factorial *p* values are from the blocked 2 × 5 ANOVA model. Within columns with a significant H × F interaction (tapped bulk density, WAC, and swelling power), means sharing at least one superscript letter are not significantly different (Tukey HSD, *p* < 0.05). For bulk density and OAC, the interaction was nonsignificant; interpretation should focus on the significant fermentation‐duration main effect.

Abbreviations: 0HT = non‐thermally pretreated okara, F = fermentation duration, H = thermal pretreatment, HT = thermally pretreated okara, OAC = oil absorption capacity, WAC = water absorption capacity.

Bulk density varied within a narrow range (0.375–0.437 g/cm^3^), indicating modest changes in loose‐packing behavior. Its significant fermentation‐duration effect, with nonsignificant thermal and interaction effects, points primarily to fermentation‐related variation. Tapped bulk density showed a clearer response, ranging from 0.518 ± 0.015 g/cm^3^ in the non‐thermally pretreated 0‐h sample to 0.613 ± 0.021 g/cm^3^ at 12 h. The significant interaction indicates that particle packing under tapping changed differently with fermentation depending on pretreatment. Fermentation and other processing treatments have been shown to modify the structure and physicochemical characteristics of okara and its dietary‐fiber matrix (Lei et al. 2025; Meng et al. 2025). The interaction observed in the present study may therefore reflect treatment‐dependent changes in the accessibility and organization of water‐binding components, although protein conformation, fiber fractions, and microstructure were not measured.

Water absorption showed the strongest functional response. WAC ranged from 3.75 ± 0.04 to 5.02 ± 0.20 g/g, with the thermally pretreated 24‐h sample recording the highest value. The significant interaction and nonsignificant thermal main effect show that pretreatment did not uniformly increase WAC; rather, it modified the response to fermentation duration. Okara contains both dietary fiber and protein that contribute to water binding. Lian et al. (2020), for example, reported a WAC of 8.29 g/g in soy okara flour containing 57.0 g/100 g insoluble fiber and only 3.41 g/100 g starch and found that okara increased batter viscosity and moisture retention in gluten‐free bread. Thermal processing can alter soy‐protein conformation, while fermentation can modify the structural and physicochemical characteristics of the okara matrix (Chen et al. 2025; Meng et al. 2025). The observed interaction may therefore reflect treatment‐dependent changes in the accessibility and organization of water‐binding components rather than a simple progressive effect of either treatment.

Absolute WAC was lower than the 8.29 g/g reported by Lian et al. (2020), consistent with differences in composition, processing history, particle characteristics, and analytical procedure. Even so, 3.75–5.02 g/g indicates substantial water binding. Higher WAC may aid moisture retention or viscosity development but can also increase water demand during processing.

OAC ranged from 2.98 ± 0.11 to 3.71 ± 0.48 g/g and was primarily influenced by fermentation duration, with no evidence that thermal pretreatment altered the response. Oil absorption in protein‐ and fiber‐rich materials may reflect both physical oil retention and interactions with nonpolar regions of macromolecules. Heat treatment can modify soy‐protein structure and functionality (Chen et al. 2025), while fermentation can alter the structural and physicochemical characteristics of the okara dietary‐fiber matrix (Meng et al. 2025). These treatment‐related structural changes provide a plausible basis for the nonlinear WAC response observed in the present study.

Swelling power ranged from 2.73 ± 0.06 to 3.17 ± 0.06 g/g and showed significant effects of thermal treatment, fermentation duration, and interaction. Non‐thermally pretreated samples reached their lowest value at 36 h, then partially recovered at 48 h, whereas the thermally pretreated series followed a different trajectory. The significant interaction indicates that prior heating modified the subsequent response of the flour matrix to hydration and heating. Because okara contains relatively little starch but is rich in insoluble dietary fiber and protein (Lian et al. 2020), its swelling behavior may not be attributable solely to starch gelatinization. Processing‐induced changes in the structure and physicochemical properties of okara and its dietary‐fiber matrix have been reported (Lei et al. 2025; Meng et al. 2025), providing a plausible basis for contributions from fiber and protein to the observed swelling response.

Hausner ratio (1.32–1.43) and Carr's index (23.92%–30.03%) remained statistically unchanged despite treatment‐related changes in bulk and tapped density. The treatments therefore did not measurably alter relative compressibility as reflected by these indices. Because Hausner ratio and Carr's index are influenced by powder characteristics and measurement conditions and may not reliably represent flow behavior across different powders, they are more appropriately interpreted comparatively in the present study (Düsenberg et al. 2023).

Functional performance alone did not identify the most suitable treatment condition. The thermally pretreated 24‐h sample recorded the highest WAC (5.02 ± 0.20 g/g) but occurred beyond the fermentation interval associated with low beany‐flavor intensity. In contrast, the thermally pretreated 12‐h sample combined low beany‐flavor intensity with a WAC of 4.16 ± 0.03 g/g, OAC of 3.59 ± 0.07 g/g, and swelling power of 2.87 ± 0.06 g/g. This treatment therefore provided a practical balance between sensory and functional characteristics rather than representing an optimum based on a single response or evidence of consumer acceptance.

### 3.7 Multivariate Relationships Among Principal Quality Attributes

Principal component analysis (PCA) was used to explore relationships among selected physicochemical, nutritional, phytochemical, functional, color, and sensory characteristics (Figure 4). Standardized treatment means for pH, crude protein, total phenolic content (TPC), phytate, water absorption capacity (WAC), swelling power (SP), instrumental lightness (*L*), and beany‐flavor intensity were included. The first three principal components explained 81.10% of the total variation, comprising 43.56% for PC1, 22.82% for PC2, and 14.72% for PC3. PC1 and PC2 together accounted for 66.38%. Because the PCA was based on 10 treatment profiles and eight variables, it was interpreted as an exploratory description of multivariate structure rather than an inferential test of treatment differences.

**FIGURE 4:**
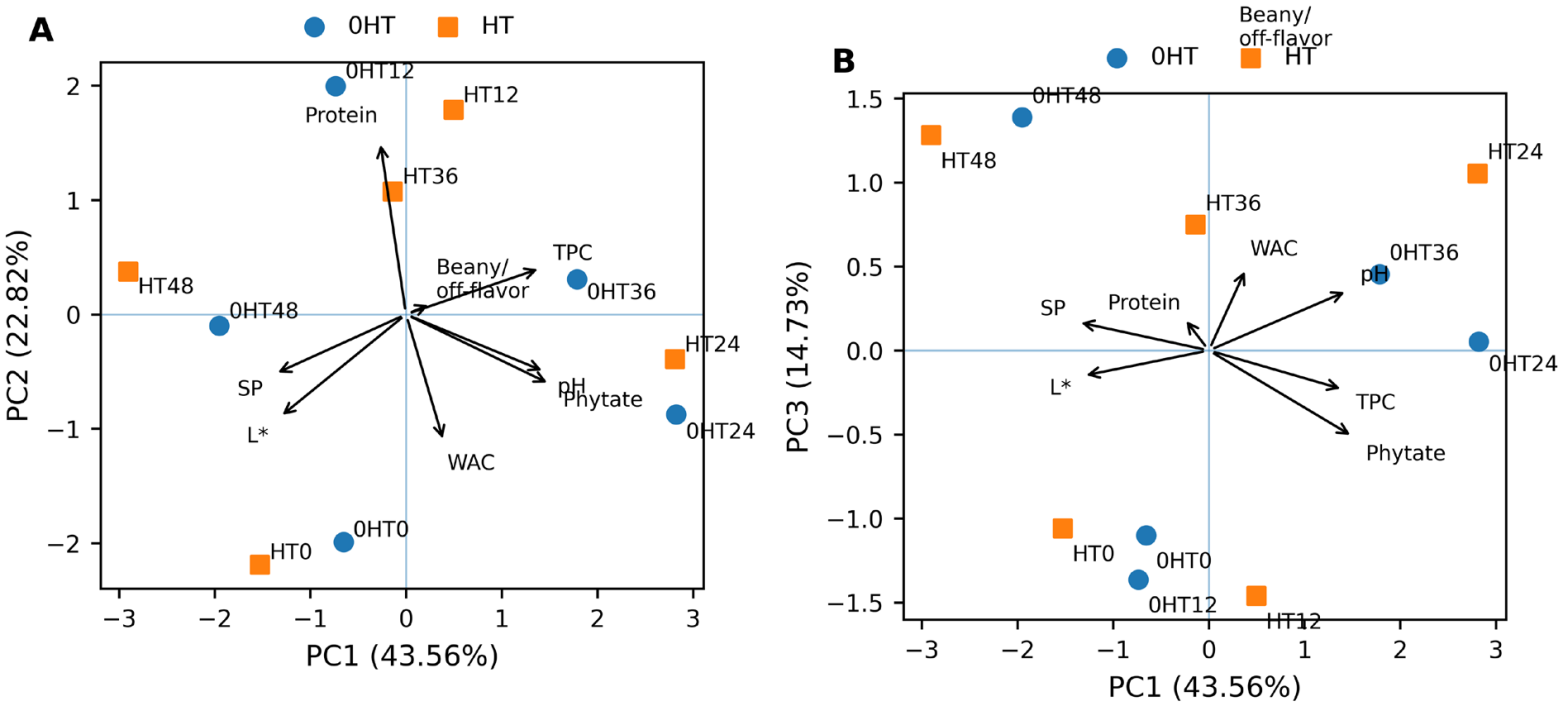
Principal component analysis biplots of selected quality attributes of okara flour subjected to thermal pretreatment and spontaneous fermentation. (A) PC1 versus PC2 and (B) PC1 versus PC3. PCA was performed on standardized treatment means for pH, crude protein, total phenolic content (TPC), phytate, water absorption capacity (WAC), swelling power (SP), instrumental lightness (L*), and beany‐flavor intensity. PC1, PC2, and PC3 explained 43.56%, 22.82%, and 14.72% of total variance, respectively. 0HT = non‐thermally pretreated okara; HT = thermally pretreated okara. Numbers following treatment codes indicate fermentation duration (h)

PC1 showed strong positive loadings for phytate (0.876), pH (0.845), and TPC (0.821), whereas SP (−0.798) and *L* (−0.767) loaded in the opposite direction. The 24‐h treatments occupied the positive PC1 region, consistent with their comparatively high pH, TPC, and phytate values, while the 48‐h treatments were positioned mainly toward the negative region. These opposing loadings indicate that the physicochemical, phytochemical, and functional attributes did not vary in parallel across treatments, consistent with the nonlinear patterns observed in the univariate analyses.

PC2 was associated mainly with crude protein (loading 0.878), whereas WAC loaded in the opposite direction (−0.650). The 12‐h treatments were positioned mainly in the positive PC2 region, consistent with their comparatively high measured protein contents, while the unfermented treatments occurred toward the negative region. This opposition reflects the contribution of these variables to treatment separation within the multivariate dataset and does not imply a direct mechanistic relationship between protein content and WAC.

Beany‐flavor was represented most strongly by PC3. Its loading was weak on PC1 (0.167) and PC2 (0.059) but strong on PC3 (0.956). Unfermented and 12‐h treatments were positioned mainly on the negative side of PC3, whereas treatments fermented for 24–48 h shifted toward the positive region. This pattern was consistent with the factorial sensory analysis, in which beany‐flavor intensity remained low through 12 h and increased thereafter. The principal sensory change associated with prolonged fermentation therefore occurred largely along a dimension distinct from the dominant physicochemical and compositional variation represented by PC1 and PC2.

The PCA further illustrated that prolonged fermentation altered quality attributes in different directions rather than producing a uniform shift across responses. The thermally pretreated 12‐h sample had the lowest observed beany‐flavor intensity and was positioned in a region associated with comparatively high measured protein content. However, because the PCA was based on only 10 treatment profiles, it was used as an exploratory description of multivariate relationships and not as evidence of statistical superiority among treatments. The factorial analysis remains the basis for statistical inference, and neither the thermal main effect nor the thermal pretreatment × fermentation‐duration interaction was significant for beany‐flavor intensity. The multivariate pattern therefore complements, rather than replaces, the univariate findings and is consistent with the first 12 h of fermentation as a practical treatment window.

## 4 Conclusion

Thermal pretreatment and spontaneous fermentation affected several quality characteristics of okara flour, although the magnitude and direction of the responses varied across attributes. Fermentation duration was the principal factor affecting beany‐flavor intensity, whereas the thermal main effect and thermal pretreatment × fermentation duration interaction were nonsignificant. Beany‐flavor intensity was lowest during the early fermentation period, with the lowest observed mean recorded for the thermally pretreated 12‐h sample, while sensory color and texture remained comparatively stable despite treatment‐related changes in instrumental color.

Thermal pretreatment altered several fermentation responses, but conditions yielding favorable functional or compositional values did not always coincide with the lowest beany‐flavor intensity. Among the conditions evaluated, thermal pretreatment at 90°C–100°C for 5 min, followed by approximately 12 h of spontaneous fermentation, was the most favorable processing condition for maintaining low beany‐flavor intensity while preserving the broader quality characteristics assessed. The selected treatment is a practical multi‐response option within the experimental conditions and does not imply statistical superiority or a universally optimal treatment.

The sensory findings reflect attribute intensity rather than consumer liking and therefore do not establish consumer acceptance. Likewise, reductions in phytate do not demonstrate improved mineral bioavailability. The microbiological safety of the spontaneously fermented okara was not verified, and such verification would be necessary before practical food application, particularly given the 24‐h pH rebound and the use of an uncharacterized natural microbial population without a starter culture. Further work should characterize the microorganisms, metabolites, and flavor compounds associated with spontaneous fermentation and evaluate the selected processing condition through microbiological safety assessment, mineral bioaccessibility, protein digestibility, and consumer testing in formulated foods.

## Author Contributions

**Abdul‐Aziz Bawa:** conceptualization, investigation, writing – original draft, methodology, data curation, formal analysis, writing – review and editing, validation, visualization. **Fortune Akabanda:** validation, visualization, writing – review and editing. **Newlove Akowuah Afoakwah:** visualization, supervision, validation, writing – review and editing.

## Funding

The authors have nothing to report.

## Conflicts of Interest

The authors declare no conflicts of interest.

## Data Availability

The data that support the findings of this study are available from the corresponding author upon request.
